# Human umbilical cord mesenchymal stem cells combined with pirfenidone upregulates the expression of RGS2 in the pulmonary fibrosis in mice

**DOI:** 10.1186/s12931-022-02192-6

**Published:** 2022-10-01

**Authors:** Xian Wu, Hao Gou, Ou Zhou, Huijun Qiu, Hanmin Liu, Zhou Fu, Lina Chen

**Affiliations:** 1 Division of Pediatric Pulmonology and Immunology, West China Second University Hospital, Sichuan University, Chengdu, 610041 Sichuan China; 2 Key Laboratory of Birth Defects and Related Diseases of Women and Children, Sichuan University, Ministry of Education, Chengdu, 610041 Sichuan China; 3 NHC Key Laboratory of Chronobiology, Sichuan University, Chengdu, 610065 Sichuan China; 4 Hospital of Chengdu University of Traditional Chinese Medicine, Chengdu, 610075 Sichuan China; 5 Pediatric Research Institute, Ministry of Education Key Laboratory of Child Development and Disorders, Children’s Hospital of Chongqing Medical University, Chongqing, 400015 China; 6 Department of Respiratory Medicine, Children’s Hospital of Chongqing Medical University, Chongqing, 400015 China; 7 Chongqing Engineering Research Center of Stem Cell Therapy, Chongqing, 400015 China

## Abstract

**Objective:**

The therapeutic effect of umbilical cord-derived mesenchymal stem cells (hUC-MSCs) in combination with pirfenidone (PFD) on pulmonary fibrosis in mice and its possible mechanism were investigated.

**Methods:**

C57BL/6 mice were randomly divided into six groups: control group, model group, P_10_ group, P_30_ group, P_100_ group, and P_300_ group. Modeled by tracheal intubation with 3 mg/kg bleomycin drip, each dose of PFD was administered daily by gavage from day 7 onwards. The mice were observed continuously for 21 days and survival was recorded. Lung tissues were collected on day 21, and hematoxylin–eosin (HE) and Masson staining were performed to assess morphological changes and collagen deposition in the lungs. Collagen content was measured by the Sircol method, and fibrosis marker levels were detected by PCR and Western blot. Another batch of C57BL/6 mice was then randomly divided into five groups: hUC-MSC control group, model group, P_100_ group, hUC-MSC treatment group, and hUC-MSCs + P_30_ group. On day 7, 5 × 10^5^ hUC-MSCs were injected into the tail vein, the mice were administered PFD gavage daily from day 7 onwards, and their survival was recorded. Lung tissues were collected on day 21 to detect pathological changes, the collagen content, and the expression of regulator of G protein signaling 2 (RGS2). Pulmonary myofibroblasts (MFBs) were divided into an MFB group and an MFB + hUC-MSCs group; different doses of PFD were administered to each group, and the levels of RGS2, intracellular Ca^2+^, and fibrosis markers were recorded for each group.

**Results:**

Compared with other PFD group doses, the P_100_ group had significantly improved mouse survival and lung pathology and significantly reduced collagen and fibrosis marker levels (*p* < 0.05). The hUC-MSCs + P_30_ group had significantly improved mouse survival and lung pathology, significantly reduced collagen content and fibrosis marker levels (*p* < 0.05), and the efficacy was better than that of the P_100_ and hUC-MSCs groups (*p* < 0.05). RGS2 expression was significantly higher in the MSCs + P_30_ group compared with the P_100_ and hUC-MSCs groups (*p* < 0.05). PFD increased RGS2 expression in MFBs (*p* < 0.05) in a dose-dependent manner. Compared with PFD and hUC-MSCs treatment alone, combination of hUC-MSCs and PFD increased RGS2 protein levels, significantly decreased intracellular Ca^2+^ concentration, and significantly reduced fibrosis markers.

**Conclusion:**

The findings suggest that hUC-MSCs combined with low-dose PFD have a therapeutic effect better than that of the two treatments used separately. Its effect on attenuating bleomycin-induced pulmonary fibrosis in mice is related to the increase of RGS2.

**Supplementary Information:**

The online version contains supplementary material available at 10.1186/s12931-022-02192-6.

## Background

Idiopathic pulmonary fibrosis (IPF), a diffuse inflammatory disease of the lower respiratory tract with unknown etiology, is a chronic interstitial lung disease characterized by progressive dyspnea and deterioration of lung function [1]. Its incidence increases every year; the median survival is only 2.5–3.5 years, and the prognosis is worse than that of many types of cancer [2, 3].

The 2015 edition of the clinical practice guidelines for the treatment of IPF states that the use of prednisone, azathioprine, N-acetylcysteine monotherapy, anticoagulants, platelet-derived growth factor receptor antagonists, endothelin receptor antagonists, and 5-phosphodiesterase inhibitors is strongly discouraged in patients with IPF, and that there are only four conditional recommended drugs, including pirfenidone (PFD), nintedanib, N-acetylcysteine combination therapy, and antacid therapy [1]. Although the emergence of PFD has benefited many IPF patients, in the 2015 edition of the clinical practice guidelines for the treatment of IPF, PFD was defined as a conditionally recommended drug due to its high effective dose, numerous adverse effects, and effectiveness only in mild-to-moderate IPF [1, 4, 5]. Therefore, the current monotherapy for PFD is not effective, so it is important to find an improved treatment.

Cell therapy based on stem cell technology has been a popular trend in recent years [6]. Human umbilical cord-derived mesenchymal stem cells (hUC-MSCs) are a type of adult stem cell with multi-directional differentiation potential derived from the mesoderm, and they are widely used because of the simplicity of their extraction, lack of ethical restrictions, strong immunomodulatory properties, and low immunogenicity [7, 8]. Clinical trials on hUC-MSCs for the treatment of various systemic diseases have been conducted worldwide, confirming the safety of hUC-MSCs in clinical applications [9–11]. Recent studies have shown that hUC-MSCs induce immune modulation and inflammation control through paracrine secretion of multiple factors and microvesicles, which can reduce profibrotic factors and collagen deposition, producing therapeutic effects in animal models of bleomycin-induced early pulmonary fibrosis [12, 13]; however, hUC-MSCs offer limited therapeutic effects in the late stage of pulmonary fibrosis [14].

RGS2 is a suppressor of G protein-coupled proteins that has received increasing attention in fibrotic diseases [15–17]. Recent studies have found that RGS2 expression is significantly downregulated in animal models of renal fibrosis and cardiac hypertrophy, suggesting that RGS2 plays an important role in regulating the pathogenesis of fibrosis [18, 19]. Enhanced RGS2 expression protects against pulmonary fibrosis in mice, while knockdown of RGS2 promotes fibrosis in mice, which demonstrates that endogenous RGS2 has an antifibrotic function and that RGS2 is the basis of the antifibrotic effect of PFD [20]. However, the biological function of RGS2 in bleomycin-induced pulmonary fibrosis in mice and its mechanism of action are rarely reported in the literature.

In this study, we established a mouse model of pulmonary fibrosis using bleomycin to investigate the effects of hUC-MSCs combined with low-dose PFD by observing the survival rate and lung pathological changes and by detecting the expression level of pulmonary fibrosis markers in the mice. PFD and hUC-MSCs were combined to act on myofibroblasts (MFBs) to observe the effect of their combination on the expression of RGS2, which has antifibrotic effects, to preliminarily explore the possible mechanism of the combined treatment and provide a theoretical basis for the treatment of IPF with hUC-MSCs combined with PFD.

## Methods

### Animal grouping and treatment

SPF-grade 7-week-old C57BL/6 male rats were purchased from the Animal Experiment Center of Chongqing Medical University and housed in an SPF-grade breeding room at 22–26 ℃, 55–60% humidity, and 12 h/day light rotation; modeling was started after 1 week of adaptation.

We divided 60 C57BL/6 rats into 6 groups using the random number method: control group (N), bleomycin model group (B), P_10_ group (10 mg/kg PFD, P_10_), P_30_ group (30 mg/kg PFD, P_30_), P_100_ group (100 mg/kg PFD, P_100_), and P_300_ group (300 mg/kg PFD, P_300_). The pulmonary fibrosis model was established by dripping bleomycin 3 mg/kg into the lungs of mice at an equal rate through tracheal intubation, and the control group was administered an equal amount of saline at an equal rate. After successful modeling, each PFD dose group was administered 10 mg/kg, 30 mg/kg, 100 mg/kg, or 300 mg/kg PFD (Adamas Reagents, China) suspension by gavage from day 7 after modeling, and the control group was administered an equal volume of saline once per day until day 21 after modeling.

Another batch of 60 C57BL/6 mice was divided into 6 groups using the random number method: control group (N), hUC-MSCs control group (N + M), bleomycin model group (B), hUC-MSCs treatment group (B + M), P_100_ group (100 mg/kg PFD, P_100_), and hUC-MSCs + P_30_ group (hUC-MSCs + 30 mg/kg PFD, B + M + P_30_). Modeling was performed by tracheal intubation with 3 mg/kg bleomycin drip, and on day 7 after modeling, hUC-MSCs control and hUC-MSCs treatment groups were injected with 5 × 10^5^/200 μL P_4_ generation hUC-MSCs via the tail vein of mice, and the non-MSC group was injected with an equal amount of saline. Starting from day 7 after modeling, the P_100_ group and the hUC-MSCs + P_30_ group (B + M + P_30_) were administered 100 mg/kg and 30 mg/kg PFD suspension by gavage, respectively, and the control group was administered an equal volume of saline once daily until day 21 after modeling. The survival of the mice was recorded, and lung tissues were collected on day 21. The mice survival curves, analysis of lung histopathology, determination of lungs collagen content, detection of mRNA, markers of lung fibrosis, and detection of RGS2 expression will be described briefly.

### Survival curves

The survival of each group of mice was observed and recorded, and survival curves were plotted using GraphPad Prism 5.0.

### Lung histological analysis

Paraformaldehyde-fixed mouse lung sections were analyzed with hematoxylin–eosin (HE) or Masson’s trichrome staining (Leagene Biotechnology, China) to assess fibrotic changes in the lungs. Three anterior, middle, and posterior sections of each lung specimen of mice were harvested for HE and Masson staining, and five high-magnification views of each section were then selected for observation and scored separately using the modified Ashcroft method (scale range 0 to 8) [21]. The histopathological score of pulmonary fibrosis for each mouse was expressed as the mean score of the sections.

### Determination of intrapulmonary collagen content

We took 80 mg of lung tissue from the right lung according to the Sircol method (Biocolor, UK) for measuring soluble collagen. A standard curve was produced using collagen standards, and the collagen concentration was calculated from the standard curve. Soluble collagen content was calculated according to the following formula: soluble collagen content = calculated collagen concentration × total volume of hydrolysate (1 mL)/80 mg × total wet weight of right lung tissue × 1000 (μg).

### Fluorescence quantitative PCR

Total RNA was extracted from each group of lung tissues using the TRIZOL method, and the RNA was reverse-transcribed into cDNA using a reverse transcription kit (Takara, Japan). The primers, including β-actin, type I collagen a1 (Col1a1), type I collagen a2 (Col1a2), α-smooth muscle actin (α-SMA), RGS2, calcium adhesion protein E (E-cad), and fibronectin (FN) were synthesized by Chengdu Kengke Zixi Biotechnology Co. Ltd. The cDNA was used for fluorescence quantitative PCR, and the expression of Col1a1, Col1a2 and α-SMA, RGS2, E-cad, and FN was detected using the β-actin gene as the internal reference gene level. The genes, mRNA, and protein are referred to using official gene symbols, as provided by the National Center for Biotechnology Information (NCBI; https://www.ncbi.nlm.nih.gov).

### Western blot

Protein was extracted from lung tissue using RIPA lysis buffer (Beyotime Biotechnology, China). Samples were electrophoresed and subjected to Western blot analysis using a primary antibody against RGS2 (Santa Cruz Biotechnology, RGS2 BC-43, sc-100761) and β-actin. Anti-mouse secondary antibodies (ZSGB Biotechnology, China) were used to capture images with chemiluminescence and fluorescence systems (Syngene U.S.).

### Acquisition and identification of hUC-MSCs

P_2_ generation hUC-MSCs were obtained from Chongqing Stem Cell Therapy Engineering Technology Research Center. The hUC-MSCs were cultured and identified according to the methods reported in the literature [22, 23]. The hUC-MSCs were grown to P_4_ generation; cell suspensions were collected after trypsin digestion, centrifuged, and resuspended with PBS and counted to a final cell concentration of 2.5 × 10^6^/mL then placed on ice for use.

### Acquisition, grouping and treatment of myofibroblasts

P_4_ generation NIH3T3 and HLF-9 were spread in six-well plates (Corning, USA) at 1 × 10^6^ in a humidified incubator at 37 °C with 5% CO2. When the cell fusion reached approximately 80%, they were replaced with fresh DMEM (Gibco, USA) medium containing 1% FBS (Gibco, USA), treated with 4 ng/ml transforming growth factor-β1 (TGF-β1, Peprotech USA), and cultured for 24 h (i.e., MFBs of both cell lines). Different concentrations of PFD were added to the MFBs: 0, 2, 4, 6, 8, and 10 mM. The cells in each group were collected after 2 h of incubation, and RGS2 mRNA expression was detected by RT-PCR to determine the effective increase in concentration of RGS2 mRNA caused by PFD. The effective concentration of PFD was added to the MFBs separately, the cells in each group were collected separately after 24 h of culture, and the mRNA expression of fibrosis markers was detected by RT-PCR.

The cells were divided into three groups according to different culture methods: NIH3T3 group (N + T) or HLF-9 group (H + T), hUC-MSCs group (M + T), and NIH3T3 + hUC-MSCs group (N + T + M) or HLF-9 + hUC-MSCs group (H + T + M). NIH3T3 or HLF-9 were cultured in the lower chamber of one well of the six-well co-culture plate alone (N or H). The P_4_ generation hUC-MSCs were cultured in the upper chamber of the other well of the six-well co-culture plate (M + T). MFBs were cultured in the lower chamber of the six-well co-culture plate, and P_4_ generation hUC-MSCs were cultured in the upper chamber of the six-well co-culture plate (N + T + M or H + T + M). All cells were cultured using DMEM/F12 (Gibco, USA) containing 5% FBS. When the cell fusion of each group reached approximately 80%, the effective concentration of PFD or equal amount of PBS buffer was added, each group of cells was collected after 24 h of culture, and the mRNA expression of RGS2 and fibrosis markers was detected by RT-PCR.

The hUC-MSCs were divided into three groups: hUC-MSCs group (M), hUC-MSCs + TGF-β1 group (M + T), and hUC-MSCs + TGF-β1 + PFD group (M + T + P). The hUC-MSCs were cultured using DMEM/F12 containing 5% FBS, and when the fusion of cells in each group reached approximately 80%, they were replaced with fresh DMEM containing 1% DMEM medium with FBS, treated with 4 ng/ml TGF-β1, and cultured for 24 h. Cells in the M + T group were collected and detected by RT-PCR for E-cad, FN, and α-SMA mRNA expression. In the M + T + P group, PFD or an equal amount of PBS buffer was added after 24 h of TGF-β1 treatment, and the cells were collected after 24 h of culture and detected by RT-PCR for E-cad, FN, and α-SMA mRNA expression.

### Measurement of intracellular Ca^2+^

HLF-9 and NIH3T3 seeded into 6-well plates at 1 × 10^4^cells/well and treated with or without PFD and hUC-MSCs were removed from the incubator. Culture solution was removed, the cells were rinsed 3 times with HEPES and 5 mM Fluo-3/AM (Beyotime Biotechnology, China) according to kit instructions was added. Next, HLF-9 and NIH3T3 were incubated at 37 °C for 40 min, followed by 3 washes with HEPES to discard the remaining extracellular probe and HEPES culture medium was used to cover all cells in the troughs. Laser scanning confocal microscopy (LSCM) was performed to observe the fluorescence intensity (FI) of the cells, using an excitation wavelength of 488 nm and emission wavelength of 530 nm.

### RNA interference

We used interfering RNA (siRNA) to examine the functional consequences of downregulation of endogenous RGS2. The siRNA for RGS2 was previously generated with AAGGAAAATATACACCGACTT as target sequence for the RNA interference experiments [24]. Confusing siRNA sequences derived from the designed siRNA sequences were used as negative controls. The siRNA sequence was synthesised by Shanghai Tuo Ran Biological Technology Co., Ltd. NIH3T3 was inoculated in 6-well plates one day before transfection, and complete medium (DMEM/F12 with 10% FBS) was added to each well. Before transfection, siRNA and lipfectmaine^TM^2000(lip2000, Invitrogen, USA) were diluted separately with serum-free medium Opti-MEM® I (Gibco, USA) and then gently mixed and incubated for 20 min at room temperature. The siRNA-lip2000 mixture was added to NIH3T3 in 6-well plates and incubated under normal conditions (37 °C and 5% CO_2_) for 6 h. The medium containing siRNA-lip2000 mixture in the wells was removed and replaced with complete medium to continue incubation under normal conditions. One group of cells was used to detect cell activity, and the other group was cultured for 48 h. Cells were collected first for counting and then protein extraction, and the expression level of RGS2 within siRNAs and controls were used to detect by Western blot and QT-PCR.

### Statistical analysis

Data are expressed as means ± SEM. Comparisons between groups were made using Student's t test for unpaired observations or two-factor ANOVA and Bonferroni correction for multiple comparisons. *p* < 0.05 was considered statistically significant.

## Results

### Therapeutic effects of different doses of PFD in BLM induced pulmonary fibrosis mice

**Fig. 1:**
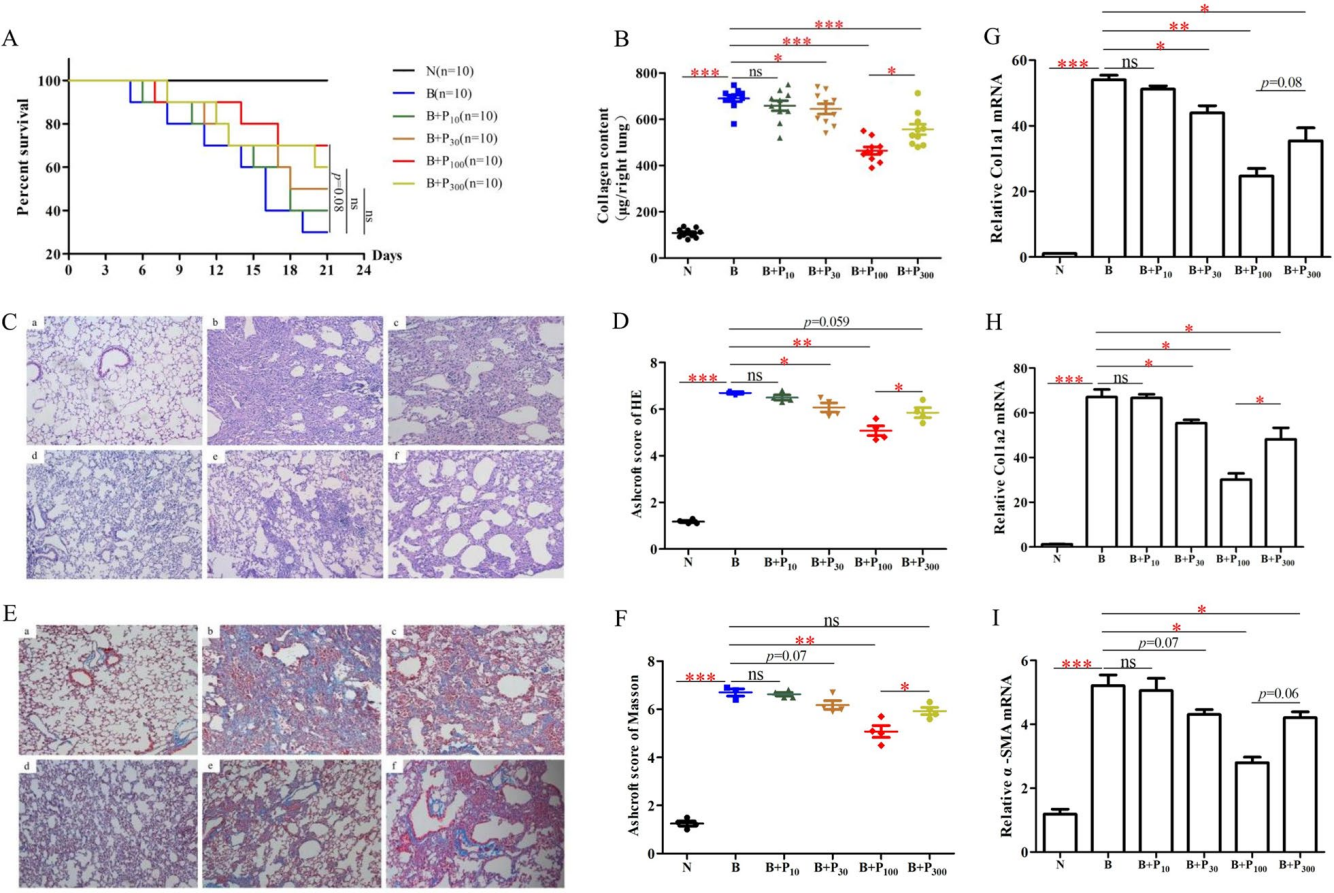
Therapeutic effects of different doses of PFD in bleomycin (BLM)-induced pulmonary fibrosis in mice. **A** The Kaplan–Meier survival curves of different doses of PFD groups (n = 10 each group). **B** The total soluble collagen content in the right lungs of different groups of mice (μg/right lung) (n = 10). **C**, **E** Representative pictures of hematoxylin–eosin (HE) and Masson staining of lung sections of different groups of pulmonary fibrosis mice. Scale bars: 200 μm. **D**, **F** The Ashcroft score of HE and Masson staining of different groups of pulmonary fibrosis mice (n = 3–4). **G**, **H**, **I** The mRNA levels of main pulmonary fibrosis markers (Col1a1, Col1a2, and α-SMA) of different groups of mice. Values are means ± SEM (n = 3). *p < 0.05; **p < 0.01; ***p < 0.01

Compared with the survival time in the model group (B), the low dose of PFD (10 mg/kg, P_10_) alone could not prolong the survival time of mice with pulmonary fibrosis, and there was a trend to prolong the survival time in the low-dose group (30 mg/kg, P_30_), the medium-dose group (100 mg/kg, P_100_), and the high-dose PFD (300 mg/kg, P_300_) alone; however, the differences were not significant (*p* = 0.38, *p* = 0.08, *p* = 0.19, respectively, n = 10 each group) (Fig. 1A). Detailed survival of mice is shown in Additional file 1: Table S1.

Using the Sircol method, we detected the soluble collagen content in the lungs of mice (n = 10), and the collagen content of the right lungs of mice in the model group was significantly higher than in the control group (*p* < 0.001). Compared with the model group, the right lung collagen content of the P_10_ group did not significantly change, the collagen content of the P_30_ group was significantly reduced (*p* < 0.05), the collagen content of the P_100_ group was significantly reduced (*p* < 0.001), and the collagen content of the P_300_ group was significantly reduced (*p* < 0.001); however, the reduction was not as great as that of the P_100_ group (*p* < 0.05) (Fig. 1B).

The pathological changes in the mice of the model group on day 21 of modeling were (n = 3–4): inflammatory cell exudation, widening of alveolar septa, formation of a ground glass-like, strip-like, grid-like structure at the base of both lungs, and cystic changes of varying sizes in some areas (i.e., honeycomb lung). Compared with the lung pathology of the model group, there was no significant improvement in the P_10_ group, a trend of improvement in the P_30_ group (*p* = 0.055), a decrease in the areas of lattice-like and honeycomb shape in the lungs of the P_100_ group, and a significant improvement of the Ashcroft score (*p* < 0.001). There was some improvement in the lung pathology of the P_300_ group (*p* = 0.059), but less than that of the P_100_ group (*p* < 0.05) (Fig. 1C, D). Masson staining colored the collagen in the lungs blue, which reflected the severity of fibrosis in the lungs. A large amount of collagen deposition in the lung was visualized microscopically in the model group. Compared with the model group, there was no significant change in intrapulmonary collagen deposition in the P_10_ group; collagen deposition was significantly reduced in the P_30_, P_100_, and P_300_ groups, but the collagen reduction in the P_30_ and P_300_ groups was not as great as that in the P_100_ group (*p* < 0.05) (Fig. 1E, F).

Col1a1, Col1a2, and α-SMA are considered the main pulmonary fibrosis markers, and their levels reflect the degree of pulmonary fibrosis [25]. Col1a1 and Col1a2 mRNA levels were significantly higher in the model group than in the control group. Compared with the model group, there was no significant change in Col1a1 mRNA in the P_10_ group, and the expression of Col1a1 mRNA was significantly reduced in the P_30_, P_100_, and P_300_ groups (Fig. 1G). Col1a2 mRNA levels were reduced in all treatment groups except for the low-dose PFD P_10_ group (*p* < 0.05, n = 3), and the most significant reduction in Col1a2 mRNA expression was observed in the P_100_ group (*p* < 0.05, n = 3). Compared with the α-SMA mRNA level in the model group, there was no significant change in the levels in the P_10_ group, a decreasing trend was observed in the P_30_ and P_300_ groups (*p* = 0.07 and *p* = 0.06, respectively, n = 3), and a significant decrease was observed in the P_100_ group (*p* < 0.05, n = 3) (Fig. 1I). Low-dose PFD (30 mg/kg) was the lowest effective dose for anti-fibrosis, medium-dose PFD (100 mg/kg) had the best efficacy among the groups with PFD alone, and high-dose PFD (300 mg/kg) was not as effective as medium-dose PFD for anti-fibrosis.

### Culture and characterization of hUC-MSCs

**Fig. 2:**
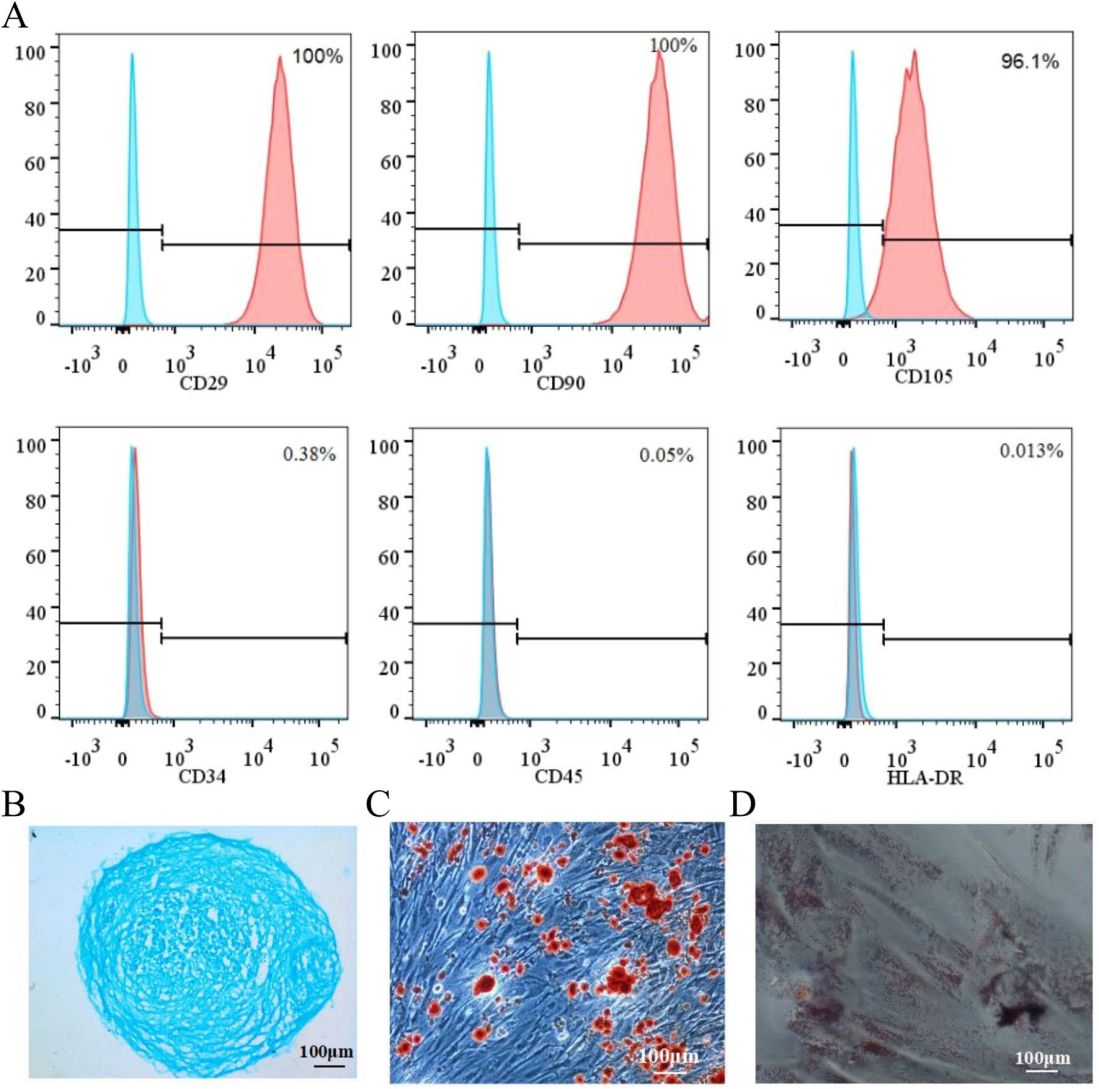
The pluripotency of hUC-MSCs. **A** The expression of the surface markers CD34, CD45, HLA-DR, CD73, CD90, and CD105 in hUC-MSCs detected by flow cytometry. **B**, **C**, **D** The results of hUC-MSC differentiation into chondrocytes, osteocytes, and adipocytes. **B** Alcian blue staining of hUC-MSCs after 24-day culture. **C** Alizarin red staining of hUC-MSCs after 20-day culture. **D** Oil Red O staining of hUC-MSCs after 15-day culture

hUC-MSCs cultured in DMEM/F12 medium at 37 °C in a 5% CO_2_ incubator were assayed for surface-specific antigens of P_4_ generation hUC-MSCs using flow cytometry. The results showed that the surface molecules CD34, CD45, and the HLA-DR positivity of hUC-MSCs were less than 2%, and CD73, CD90, and CD105 positivity were higher than 95% (Fig. 2A, Additional file 1: Fig. S1). This observation is in accordance with the standards published by the International Stem Cell Therapy Association in 2006. In addition, we examined the multidirectional differentiation potential of hUC-MSCs; our results showed that hUC-MSCs differentiated into chondrogenic (Fig. 2B, Additional file 1: Fig. S2A), osteogenic (Fig. 2C, Additional file 1: Fig. S2B), and adipogenic cells (Fig. 2D, Additional file 1: Fig. S2C).

### Therapeutic effects of hUC-MSCs combined with P_30_ in BLM induced pulmonary fibrosis mice

**Fig. 3:**
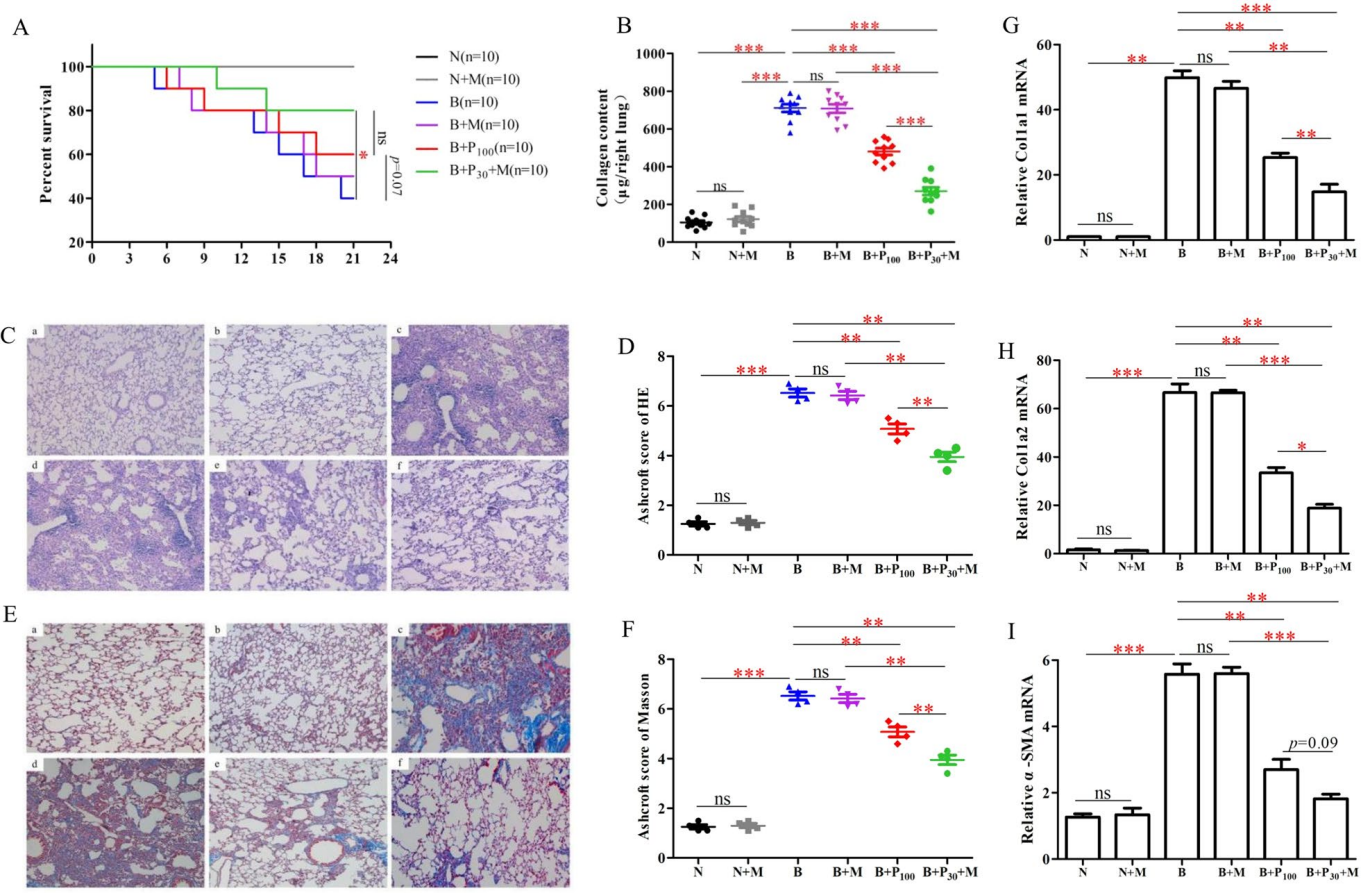
Therapeutic effects of hUC-MSCs combined with P_30_ in BLM-induced pulmonary fibrosis in mice. **A** The Kaplan–Meier survival curves of different groups (n = 10). **B** The total soluble collagen content in the right lungs of different groups of mice (μg/right lung) (n = 10). **C**, **E** Representative pictures of HE and Masson staining of lung sections of different groups of pulmonary fibrosis mice. Scale bars: 200 μm. **D**, **F** The Ashcroft score of HE and Masson staining of different groups (n = 4). **G**, **H**, **I** The mRNA levels of main pulmonary fibrosis markers (Col1a1, Col1a2, and α-SMA) in different groups of pulmonary fibrosis mice. Values are means ± SEM (n = 3).*p < 0.05; **p < 0.01;***p < 0.01

Studies have shown that hUC-MSCs can attenuate acute lung injury and early pulmonary fibrosis; however, their therapeutic effect on established pulmonary fibrosis is unclear [13–15]. Preliminary experimental data from our group showed that hUC-MSCs administered alone by transcatheter tail vein injection were less effective in treating a mouse model of pulmonary fibrosis [19, 20]; therefore, the therapeutic effect of hUC-MSCs in combination with PFD on middle- and late-stage pulmonary fibrosis is unclear. In contrast, the efficacy of high doses of PFD was also not satisfactory and was associated with significant adverse effects. Therefore, we explored the therapeutic effect of hUC-MSCs combined with the lowest effective dose of PFD (30 mg/kg, P_30_) on pulmonary fibrosis in mice. The results showed that hUC-MSCs combined with P_30_ significantly prolonged the survival time of the mice (Fig. 3A, p < 0.05, n = 10), and the survival time of the hUC-MSCs combined with P_30_ group was longer than that of the P_100_ group (*p* = 0.07). Detailed survival of mice is shown in Table. S2. The combination group had significantly reduced collagen content in the lungs (*p* < 0.001, n = 10), and the collagen content was significantly lower than that in the P_100_ group (Fig. 3B, p < 0.001). Moreover, the combination group significantly improved bleomycin-induced pulmonary lesions, with significantly better Ashcroft scores (*p* < 0.001) and significantly reduced intrapulmonary collagen deposition, all with better improvement than P_100_ (Fig. 3C–F, p < 0.01, n = 4). Regarding the expression of pulmonary fibrosis markers, Col1a1, Col1a2, and α-SMA mRNA levels were significantly decreased in the hUC-MSCs combined with the P_30_ group compared with the model group (Fig. 3G–I, p < 0.01, n = 3), where the reduced levels of Col1a1 and Col1a2 were significantly different compared to the P_100_ group alone, while the levels of α-SMA compared to the P_100_ group tended to be lower (*p* = 0.09).

### hUC-MSCs combined with low-dose P_30_ elevated RGS2 mRNA and protein expression levels in mouse lung tissue

**Fig. 4:**
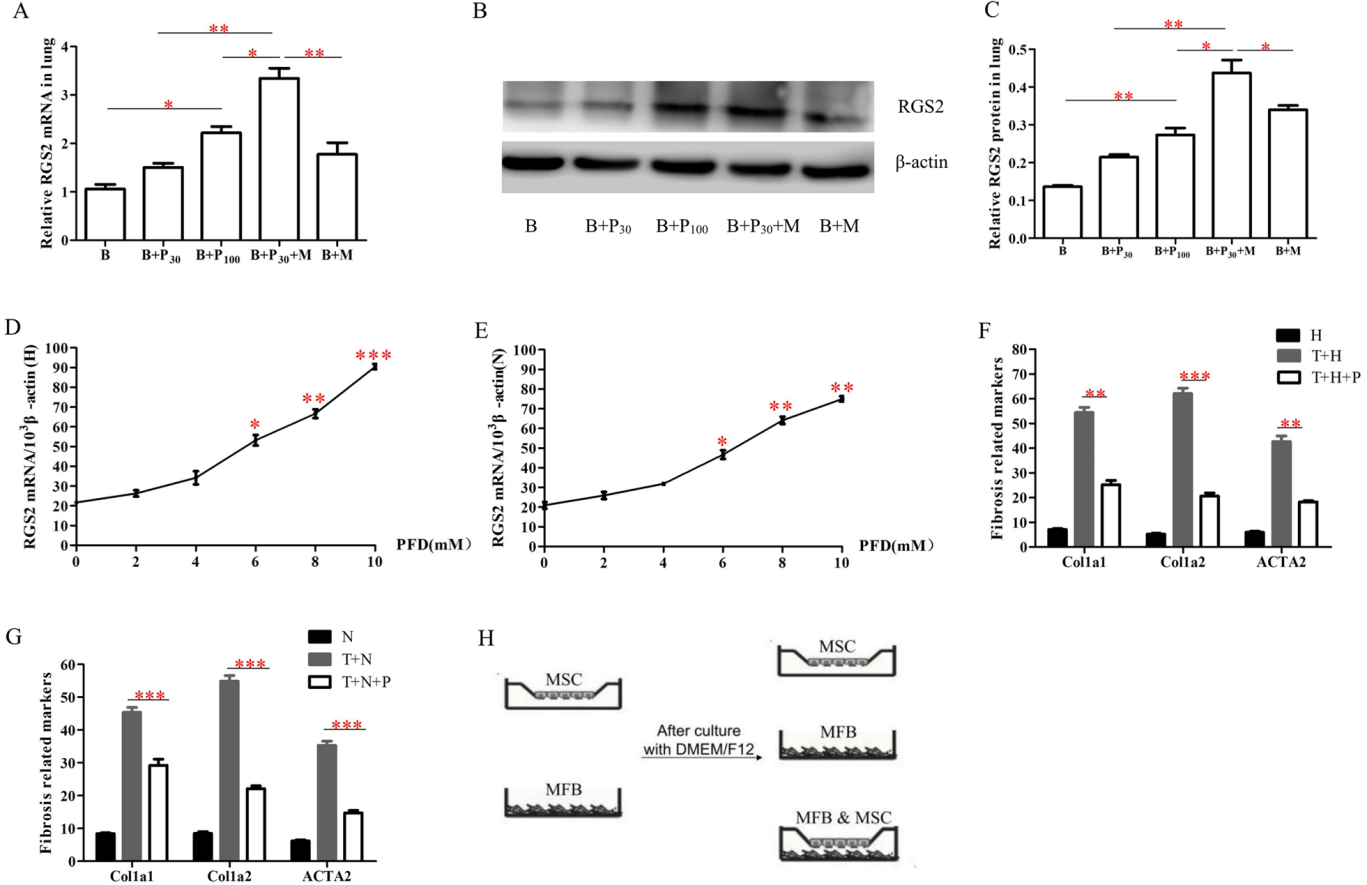
The RGS2 mRNA and protein expression levels in mouse lung tissue and cells of different groups. **A** The RGS2 mRNA levels detected by RT-PCR in mouse lung tissue of different groups (n = 3). **B** The RGS2 protein expression levels detected by Western blot in mouse lung tissue of different groups (n = 3). **C** The results are expressed as a bar graph that illustrates RGS2 protein expression, measured as the RGS2/β-actin protein ratio of these groups. The results are presented as means ± SEM (n = 3). **D**, **E** HLF-9 (**D**) and NIH3T3 (**E**) were treated with various concentrations of PFD (0–10 mM) for 2 h and were then harvested for RT-PCR analysis of RGS2 and β-actin × 1000 mRNA levels. **F**, **G** HLF-9 (**F**) and NIH3T3 (**G**) were treated with 8 mM PFD over 24 h and were then harvested for RT-PCR analysis of fibrosis marker mRNA levels (n = 3). **H** Schematic diagram of single culture and co-culture of hUC-MSCs and MFBs. *p < 0.05; **p < 0.01; ***p < 0.01

Studies have suggested that RGS2 is a novel mechanism explaining the antifibrotic effect of PFD [4]. Quantitative RT-PCR analysis in our study confirmed that, as expected, treatment of pulmonary fibrosis in mice with the optimal effective dose of PFD (100 mg/kg) increased RGS2 mRNA levels in mouse lung tissues (Fig. 4A, p < 0.05, n = 3), and RGS2 mRNA levels in the hUC-MSCs combined with P_30_ group were more elevated than those in the P_100_ group (*p* < 0.05, n = 3). Furthermore, Western blot analysis confirmed that RGS2 protein levels in lung tissue were significantly increased after PFD treatment in mice (Fig. 4B, C, n = 3), and RGS2 protein levels in the hUC-MSCs combined with P_30_ group were higher than in the P_100_ group.

### RGS2 mRNA and protein expression levels in myofibroblasts

Studies have shown that MFBs are the main cells of tissues after fibrosis [26] and that TGF-β1 induces the conversion of fibroblasts to MFBs [27]. In this experiment, two fibroblast model cells, MFBs, were obtained after stimulation of two fibroblast lines, NIH3T3 and HLF-9, for 24 h using 4 ng/mL TGF-β1. hUC-MSCs and PFD in subsequent experiments were added to fibroblasts after TGF-β1 stimulation.

Quantitative RT-PCR analysis confirmed that RGS2 mRNA was elevated in a concentration-dependent manner after PFD treatment of both MFBs (Fig. 4D, E), and the elevation of RGS2 mRNA in both MFBs treated with ≥ 6 mM PFD was significant (*p* < 0.05). As shown in Fig. 4F and G, the mRNA of fibrosis markers in both MFBs decreased significantly after 24 h of PFD treatment (n = 3).

### hUC-MSCs can affect the expression of RGS2 and the markers of pulmonary fibrosis of myofibroblasts by PFD treatment

**Fig. 5:**
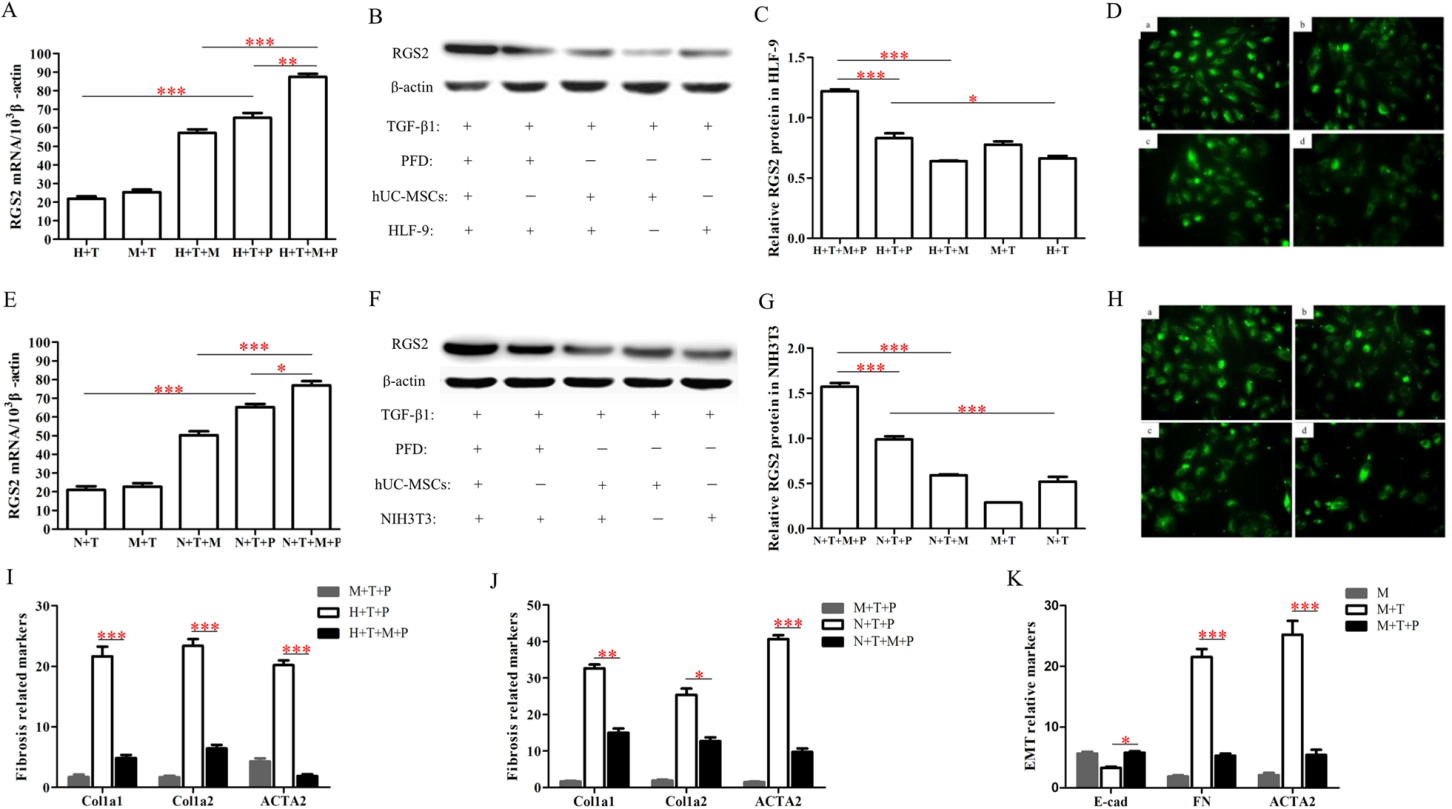
RGS2 suppresses the profibrotic effects in HLF-9 and NIH3T3. **A**, **E** HLF-9 (**A**) and NIH3T3 (**E**) were single cultured or co-cultured with hUC-MSCs 24 h after adding 4 ng/mL TGF-β1, treated with 8 mM PFD or an equal amount of PBS buffer for 2 h, and were then harvested for RT-PCR analysis of RGS2 and β-actin mRNA levels. Values are means ± SEM (n = 3). **B**, **F** RGS2 protein expression levels detected by Western blot in HLF-9 (**B**) and NIH3T3 (**E**) treated with hUC-MSCs and/or PFD. **C, G** RGS2 protein expression measured as the RGS2/β-actin protein ratio in HLF-9 (**C**) and NIH3T3 (**G**) treated with hUC-MSCs and/or PFD. Densities of bands were quantified using the ImageJ program. The results are presented as means ± SEM (n = 3). **D**, **H** HLF-9 and NIH3T3 cells were treated with TGF-β1 (4 ng/ml) firstly. Then, the Fluo-3 fluorescence of intracellular Ca^2+^ level in HLF-9 (**D**) and NIH3T3 (**H**) cells treated with PFD and/or hUC-MSCs were measured. The cells were excited at 488 nm and Ca^2+^-bound Fluo-3 emission was recorded at 530 nm. The Fluo-3 fluorescence from three separate exeachiments was plotted as means ± SEM. **I**, **J** HLF-9 (**I**) and NIH3T3 (**J**) were single cultured or co-cultured with hUC-MSCs, treated with 8 mM PFD for 24 h, and were then harvested for RT-PCR analysis of fibrosis marker mRNA levels. Values are means ± SEM (n = 3). **K** hUC-MSCs were treated with 4 ng/ml TGF-β1 and cultured for 24 h, treated with 8 mM PFD or an equal amount of PBS buffer for 24 h, and were then harvested for RT-PCR analysis of E-cad, FN, α-SMA mRNA levels. Values are means ± SEM (n = 3). *p < 0.05; **p < 0.01; ***p < 0.01

The effect of PFD (8 mM) on the expression of RGS2 and lung fibrosis markers in MFBs was explored after co-culture of hUC-MSCs with MFBs. The results showed that the differences in RGS2 mRNA levels were significant in the H + T + M + P group compared with the H + P group (Fig. 5A, p < 0.01, n = 3), as well as in the N + T + M + P group compared with the N + T + P group (Fig. 5E, p < 0.05, n = 3), indicating that hUC-MSCs further elevated RGS2 mRNA after co-action of MFB with PFD. Representative Western blot analysis of RGS2 protein expression was performed in HLF-9 (Fig. 5B) and NIH3T3 (Fig. 5F) without or with hUC-MSCs and/or PFD treatment. As shown in Fig. 5C and G, quantification of RGS2 expression is presented as means ± SEM. The results demonstrated that RGS2 protein levels were statistically significant in the H + T + M + P group compared with the H + T + P group and H + T + M group (Fig. 5C, p < 0.001), as well as in the N + T + M + P group compared with the N + T + P group and N + T + M group (Fig. 5G, p < 0.05). PFD can attenuate thrombin-induced increases in intracellular Ca^2+^ by upregulating RGS2 [4]. The intracellular Ca^2+^ increased induced by TGF-β1 [28]. In our study, intracellular Ca^2+^ levels in each group were measured, and we found that intracellular Ca2 + concentrations were reduced in the hUC-MSCs combined with PFD group (Fig. 5D, H). The Fluo-3 fluorescence intensity of intracellular Ca^2+^ in HLF-9 and NIH3T3 are shown in Additional file 1: Figs. S3 and S4 (n = 3). Co-treatment of MFBs with PFD and hUC-MSCs resulted in a significant decrease in fibrosis marker mRNA in both MFBs (Fig. 5I, J, n = 3).

Furthermore, we examined the changes in mRNA levels of endothelial–mesenchymal transition (EMT) markers in hUC-MSCs. The results showed that E-cad mRNA was significantly increased, and both FN and α-SMA mRNA expression were significantly decreased after treatment of hUC-MSCs with 8 mM PFD (Fig. 5k, p < 0.05, n = 3).

### RGS2 knockdown enhances TGF-β1-induced pro-fibrotic effects in fibroblasts.

**Fig. 6:**
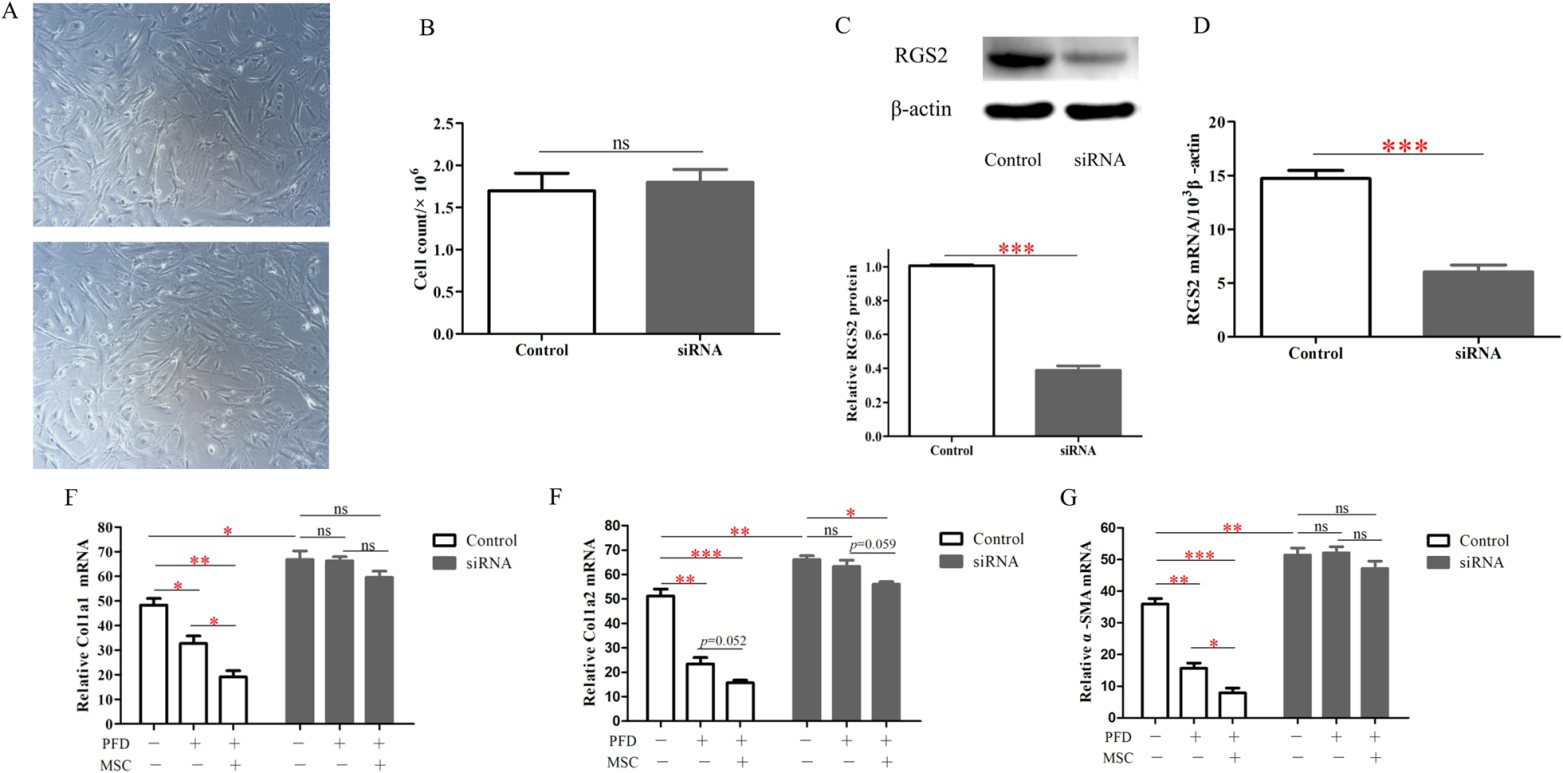
**A** Representative images of NIH3T3 transfected with or without siRNA. **B** The number of NIH3T3 in RGS2-siRNA-transfected and control groups (n = 3). **C** Top: Representative Western blot of RGS2 protein expression in Rgs2-siRNA-transfected groups and control groups of NIH3T3. Bottom: The results are expressed as a bar graph that illustrates RGS2 protein level of Western blot, measured as the RGS2/β-actin protein ratio. The results are presented as means ± SEM (n = 3). **D** The level of RGS2 mRNA was evaluated by RT-PCR presented as means ± SEM (n = 3). **F**, **G**, **H** The mRNA levels of main pulmonary fibrosis markers (Col1a1, Col1a2, and α-SMA) in Rgs2-siRNA-transfected groups and control groups of NIH3T3 without or with PFD or/and hUC-MSCs treatment. Values are means ± SEM (n = 3). *p < 0.05; **p < 0.01; ***p < 0.01

To investigate the effect of RGS2 downregulation on pulmonary fibrosis, NIH3T3 cells treated with TGF-β1 were transfected with RGS2-targeting siRNA and then cultured in vitro. Representative images of NIH3T3 transfected with or without siRNA show there are no apparent changes in cell viability (Fig. 6A). The number of NIH3T3 was almost equal between RGS2-siRNA-transfected and control groups (Fig. 6B, p > 0.05). Compared with cells transfected with control group, the RGS2 protein expression significantly decreased after transfection with RGS2-siRNA (Fig. 6C). In addition, we were able to achieve up to about 62% reduction in RGS2 mRNA (Fig. 6D).

Consistent with previous data, PFD alone treatment and hUC-MSCs in combination with PFD both reduced the increase in TGF-β1-induced fibrosis markers (Col1a1, Col1a2, and α-SMA). Importantly, reduction in RGS2 expression markedly enhanced TGF-β1-induced pro-fibrotic effects in NIH3T3 (Fig. 6E–G). In marked contrast, PFD treatment had no effect in RGS2-siRNA-NIH3T3, indicating that upregulated RGS2 is a important mediator of the anti-fibrotic effects associated with PFD treatment (Fig. 6E–G). hUC-MSCs combined with PFD.

could reduce the expression of fibrosis markers to some extent, especially Col1a2, but there was no statistical difference (*p* > 0.05).

## Discussion

Although PFD has been approved by the Food and Drug Administration (FDA) for the treatment of IPF, its therapeutic effects are limited, and its adverse effects are large. In the present study, we used hUC-MSCs combined with low-dose PFD for the treatment of pulmonary fibrosis in mice. We found that, combined with hUC-MSCs, low-dose PFD could exert antifibrotic effects superior to its threefold or even tenfold dose alone. Therefore, the findings suggest that the combination of hUC-MSCs and PFD can enhance the antifibrotic efficacy of PFD, reduce the required dosage of PFD, and decrease the incidence of adverse effects to enhance the tolerability and compliance of IPF patients.

Hisashi O et al. used concentrations of 10, 30, and 100 mg/kg PFD to treat a mouse model of bleomycin-induced pulmonary fibrosis, and the results of the study showed that the antifibrotic effect of PFD was positively correlated with the dose [29]. However, the efficacy of PFD containing higher dose gradients on pulmonary fibrosis in mice was not reported in this literature. In our present study, four dose gradients of 10, 30, 100, and 300 mg/kg PFD were applied to explore the therapeutic effects on pulmonary fibrosis and, in contrast to our expectations, the highest dose of PFD (300 mg/kg) did not achieve better antifibrotic effects than the medium dose (100 mg/kg). In the clinical trials of PFD for IPF conducted by Azuma A et al. and Wijsenbeek et al., the adverse effects of PFD included predominantly gastrointestinal reaction and were positively correlated with dose, with an incidence of 40–60%, and some patients discontinued the drug due to intolerance [30, 31]. Combined with the observation that the appetite of mice in the high-dose PFD group was poorer than in the medium- and low-dose PFD groups, it can be assumed that the PFD dose exceeded the ideal range, resulting in gastrointestinal adverse reactions and reducing the therapeutic effect of PFD. Therefore, we cannot expect to achieve a better antifibrotic effect by increasing the dose of PFD in clinical practice. For this reason, to avoid the adverse effects of higher doses of PFD, a combination of the low-dose 30 mg/kg PFD, with hUC-MSCs was used in this study instead of 100 mg/kg PFD with hUC-MSCs.

The pathogenesis of IPF remains unclear, but the main pathological changes are the proliferation and aggregation of large numbers of MFBs and the deposition of extracellular matrix, of which the main component is collagen [32]. MFBs, as the main effector cell of IPF, have a negative correlation with the severity and prognosis of IPF disease [26]. It was found that, in addition to their antifibrotic effects, hUC-MSCs have a certain degree in profibrotic effects, especially in the fibrotic environment, and can be converted to MFBs through endothelial–mesenchymal conversion [33, 34]. This may be one of the reasons for the controversial effects of hUC-MSCs in the treatment of pulmonary fibrosis. Furthermore, PFD, as a multi-cytokine inhibitor, can inhibit the expression of cytokines, such as TGF-β1, basic fibroblast growth factor (bFGF), and connective tissue growth factor (CTGF) of target cells (myo)fibroblasts, suppressing the proliferation of (myo)fibroblasts and the synthesis of collagen [35, 36]. Therefore, in this study, we examined the changes in mRNA levels of EMT markers in hUC-MSCs. The results showed that E-cad mRNA was significantly increased, and FN and α-SMA mRNA expression were both significantly decreased after treatment of hUC-MSCs with PFD. This result indicated that PFD inhibited the conversion of hUC-MSCs to mesenchymal cells. In addition, RGS2 has a negative regulatory effect on EMT and can inhibit the conversion of fibroblasts to MFBs [37]. We found that RGS2 expression increased and markers of MFBs decreased in hUC-MSCs after treatment with PFD. The experimental results indicate that PFD treatment of hUC-MSCs can reduce the conversion of hUC-MSCs to MFBs, which gives hUC-MSCs the opportunity to maximize their antifibrotic effects. This may be one of the mechanisms by which hUC-MSCs combined with PFD can achieve better efficacy compared with PFD alone.

Many studies show RGS2 plays an important role in regulating the pathogenesis of fibrosis [18, 19]. In our study, we found RGS2 increased in hUC-MSCs combined PFD mice and MFBs and we assumed that the combination of the two treatments might exert antifibrotic effects through upregulation of PFD. It has been reported that overexpression of RGS2 significantly reduces the expression of fibrosis markers and thus plays a protective role against pulmonary fibrosis [4], this result that could support our hypothesis. Previous literature reported that siRNA can knockdown the expression of endogenous RGS2 in cells [24]. In our experiments, we similarly used siRNA to knockdown RGS2 expression in NIH3T3. Our data showed that after RGS2 knockdown, PFD treatment and PFD combined with hUC-MSCs had no effect on the increase in fibrosis markers induced by TGF-β1. Interestingly, there is also evidence that PFD treatment significantly upregulated endogenous lung RGS2 expression in RGS2^+/+^ mice. More importantly, their data showed a significant increase in collagen deposition in the lungs of bleomycin-treated RGS2 knockout mice compared to that in wild-type mice [4]. Another study showed RGS2 plays a protective role and that loss of RGS2 accelerates the progression of kidney fibrosis [18]. Those all suggests that upregulating RGS2 is a key mediator of antifibrotic effects associated with PFD and/or hUC-MSCs treatment.

RGS2 is a known negative regulator of G protein signaling that inhibits the amplitude and duration of signals mediated by Gq-coupled G protein-coupled receptors (GPCRs) [38, 39]. Several Gq-coupled GPCRs and their ligands are important drivers of pulmonary fibrosis, including Gq-coupled proteinase-activated receptor 1 (PAR1), lysophosphatidic acid receptor 1, and endothelin receptor [40–43], which promote fibroblast proliferation and differentiation into an MFBs phenotype and promote the development of pulmonary fibrosis [43, 44]. Previous studies have shown that fibroblast proliferation is dependent on PAR1-mediated increases in intracellular Ca^2+^ [45, 46]. Increased intracellular Ca^2+^ concentration promotes fibroblast proliferation [27], induces fibroblast-to-MFBs conversion [47], leads to apoptosis of type II lung cells [48], and ultimately leads to the formation of pulmonary fibrosis. Therefore, GPCR-mediated Ca^2+^ signaling is closely related to IPF pathology. BLM increases Ca^2+^ signaling in MFBs in animal models [49]; in contrast, at the cellular assay level, TGF-β1 increases Ca^2+^ signaling in MFBs [50]. Therefore, reducing intracellular Ca^2+^ levels may be a promising approach to preventing or ameliorating the progression of pulmonary fibrosis. RGS2 functions as a selective modulator of Gq-mediated signaling [38, 39, 51], which can reduce intracellular Ca^2+^ levels by negatively regulating G protein signaling [4]. The results of our experiments showed that, compared with PFD and hUC-MSC treatment alone, the combination of hUC-MSCs and PFD increased RGS2 protein levels, significantly decreased intracellular Ca^2+^ concentration, and significantly reduced fibrosis markers. We found that hUC-MSC and PFD treatment significantly reduced TGF-β1-stimulated intracellular Ca^2+^ signaling which provides a molecular mechanism to explain the antifibrotic effects of RGS2.

Interestingly, our date demonstrated that RGS2 expression in hUC-MSCs and PFD was increased more than in the PFD-only treated group, and the role of hUC-MSCs in this phenomenon required further investigation. Previous studies have shown that Ca^2+^ signaling was closely associated with IPF pathology [49]. Therefore, reducing intracellular Ca^2+^ levels may be a promising approach to preventing or ameliorating the progression of pulmonary fibrosis. Li Q et al. demonstrated that hUC-MSCs could reduce intracellular Ca^2+^ concentrations [52]. PFD can attenuate thrombin-induced increases in intracellular Ca^2+^ by upregulating RGS2 [4]. We found that intracellular Ca^2+^ was significantly reduced in the hUC-MSCs combined with PFD group. Therefore, hUC-MSCs may synergize with PFD to reduce intracellular Ca^2+^ and produce stronger antifibrotic effects. Studies have shown that increased intracellular Ca^2+^ inhibits MSCs proliferation and promotes MSCs differentiation [53]. Therefore, lowering intracellular Ca^2+^ promotes MSCs proliferation and reduces MSCs differentiation, thereby maintaining more durable stem cell properties and facilitating antifibrotic effects. In addition, hUC-MSCs exert antifibrotic effects mainly through paracrine secretion [12, 13]. Therefore, we speculate that hUC-MSCs increase the expression of RGS2 in MFBs through paracrine secretion of some substances and synergize with PFD to further elevate RGS2, thereby achieving a more antifibrotic effect. More research is needed to test this hypothesis.

## Conclusion

In conclusion, the results of the present study provide direct evidence that hUC-MSCs combined with low-dose PFD had a therapeutic effect on a mouse model of pulmonary fibrosis and delayed the progression of IPF, providing new prospects for the clinical treatment of IPF. In addition, this study found that the possible mechanism of the efficacy of hUC-MSCs in combination with low-dose PFD may be related to a significant increase in the expression of the antifibrotic protein RGS2; however, the in-depth mechanism of the combined treatment should be further studied and explored.

## Supplementary Information

**Additional file 1: Table S1.** Basic data of the number of dead mice treated with different doses of PFD. **Table S2.** Basic data of the number of dead mice treated with PFD and hUC-MSCs. **Figure S1.** Flow cytometry detection of surface mesenchymal stem cell markers of hUC-MSCs. **Figure S2.** The primitive view of the differentiation of hUC-MSCs. **A**: hUC-MSCs differentiated into chondrogenic cells. **B**: hUC-MSCs differentiated into osteogenic cells. **C**: hUC-MSCs differentiated into adipogenic cells. **Figure S3.** The Fluo-3 fluorescence intensity of Ca^2+^ levels in different group of HLF-9 were shown: H + T(A), H + T + M(B), H + T + P(C); H + T + M + P(D). Values are means ± SEM (n = 3). *p < 0.05; **p < 0.01; ***p < 0.01. **Figure S4.** The Fluo-3 fluorescence intensity of Ca^2+^ levels in different group of NIH3T3 were shown: N + T(A), N + T + M(B), N + T + P(C); N + T + M + P(D). Values are means ± SEM (n = 3). *p < 0.05; **p < 0.01; ***p < 0.01.

## Data Availability

All data generated or analyzed during this study are included in this published article and its Additional file 1.
