# Supplementary material for: Human umbilical cord mesenchymal stem cells combined with pirfenidone upregulates the expression of RGS2 in the pulmonary fibrosis in mice
Source: Respir Res. 2022 Oct 1;23:270. doi: 10.1186/s12931-022-02192-6 (PMC9526322; doi:10.1186/s12931-022-02192-6)
Supplement: Supplementary file 1 — Additional file 1: Table S1. Basic data of the number of dead mice treated with different doses of PFD. Table S2. Basic data of the number of dead mice treated with PFD and hUC-MSCs. Figure S1. Flow cytometry detection of surface mesenchymal stem cell markers of hUC-MSCs. Figure S2. The primitive view of the differentiation of hUC-MSCs. A: hUC-MSCs differentiated into chondrogenic cells. B: hUC-MSCs differentiated into osteogenic cells. C: hUC-MSCs differentiated into adipogenic cells. Figure S3. The Fluo-3 fluorescence intensity of Ca2+ levels in different group of HLF-9 were shown: H + T(A), H + T + M(B), H + T + P(C); H + T + M + P(D). Values are means ± SEM (n = 3). *p < 0.05; **p < 0.01; ***p < 0.01. Figure S4. The Fluo-3 fluorescence intensity of Ca2+ levels in different group of NIH3T3 were shown: N + T(A), N + T + M(B), N + T + P(C); N + T + M + P(D). Values are means ± SEM (n = 3). *p < 0.05; **p < 0.01; ***p < 0.01. [file 12931_2022_2192_MOESM1_ESM.docx]

**Additional file 1**

**Human Umbilical Cord Mesenchymal Stem Cells Combined with Pirfenidone upregulates the Expression of RGS2 in the Pulmonary Fibrosis in Mice**

**Contents**

Table S1

Table S2

Figure S1

Figure S2

Figure S3

Figure S4

**Table S1**
**Basic data of the number of dead mice treated with different doses of PFD.**

| Days | Groups | | | | | |
| --- | --- | --- | --- | --- | --- | --- |
|  | N  (n=10) | B  (n=10) | B+P_10_  (n=10) | B+P_30_  (n=10) | B+P_100_  (n=10) | B+P_300_  (n=10) |
| 1 | 0 | 0 | 0 | 0 | 0 | 0 |
| 2 | 0 | 0 | 0 | 0 | 0 | 0 |
| 3 | 0 | 0 | 0 | 0 | 0 | 0 |
| 4 | 0 | 0 | 0 | 0 | 0 | 0 |
| 5 | 0 | 1 | 0 | 0 | 0 | 0 |
| 6 | 0 | 0 | 1 | 0 | 0 | 0 |
| 7 | 0 | 0 | 0 | 1 | 1 | 0 |
| 8 | 0 | 1 | 0 | 0 | 0 | 1 |
| 9 | 0 | 0 | 0 | 0 | 0 | 0 |
| 10 | 0 | 0 | 0 | 0 | 0 | 0 |
| 11 | 0 | 1 | 1 | 1 | 0 | 0 |
| 12 | 0 | 0 | 0 | 0 | 0 | 1 |
| 13 | 0 | 0 | 1 | 1 | 0 | 1 |
| 14 | 0 | 1 | 0 | 0 | 1 | 0 |
| 15 | 0 | 0 | 1 | 0 | 0 | 0 |
| 16 | 0 | 2 | 0 | 0 | 0 | 0 |
| 17 | 0 | 0 | 0 | 1 | 1 | 0 |
| 18 | 0 | 0 | 2 | 1 | 0 | 0 |
| 19 | 0 | 1 | 0 | 0 | 0 | 0 |
| 20 | 0 | 0 | 0 | 0 | 0 | 1 |
| 21 | 0 | 0 | 0 | 0 | 0 | 0 |

N: control group , B: bleomycin model group, P_10:_ P_10_ group (10 mg/kg PFD), P_30:_ P_30_ group (30 mg/kg PFD), P_100:_ P_100_ group (100 mg/kg PFD), P_300:_ P_300_ group (300 mg/kg PFD).

**Table S2**
**Basic data of the number of dead mice treated with PFD and hUC-MSCs.**

| Days | Groups | | | | | |
| --- | --- | --- | --- | --- | --- | --- |
|  | N  (n=10) | N+M  (n=10) | B  (n=10) | B+M  (n=10) | B+P_100_  (n=10) | B+M+P_30_  (n=10) |
| 1 | 0 | 0 | 0 | 0 | 0 | 0 |
| 2 | 0 | 0 | 0 | 0 | 0 | 0 |
| 3 | 0 | 0 | 0 | 0 | 0 | 0 |
| 4 | 0 | 0 | 0 | 0 | 0 | 0 |
| 5 | 0 | 0 | 1 | 0 | 0 | 0 |
| 6 | 0 | 0 | 0 | 0 | 1 | 0 |
| 7 | 0 | 0 | 0 | 1 | 0 | 0 |
| 8 | 0 | 0 | 0 | 1 | 0 | 0 |
| 9 | 0 | 0 | 1 | 0 | 1 | 0 |
| 10 | 0 | 0 | 0 | 0 | 0 | 1 |
| 11 | 0 | 0 | 0 | 0 | 0 | 0 |
| 12 | 0 | 0 | 0 | 0 | 0 | 0 |
| 13 | 0 | 0 | 1 | 0 | 0 | 0 |
| 14 | 0 | 0 | 0 | 1 | 0 | 1 |
| 15 | 0 | 0 | 1 | 0 | 1 | 0 |
| 16 | 0 | 0 | 0 | 0 | 0 | 0 |
| 17 | 0 | 0 | 1 | 1 | 0 | 0 |
| 18 | 0 | 0 | 0 | 1 | 1 | 0 |
| 19 | 0 | 0 | 0 | 0 | 0 | 0 |
| 20 | 0 | 0 | 1 | 0 | 0 | 0 |
| 21 | 0 | 0 | 0 | 0 | 0 | 0 |

N: control group , N+M: hUC-MSCs control group, B: bleomycin model group, B+M: hUC-MSCs treatment group, P_100:_ P_100_ group(100 mg/kg PFD),  B+M+P_30:_ hUC-MSCs+P_30_ group (hUC-MSCs+30 mg/kg PFD

**Figure S1**

**Flow cytometry detection of surface mesenchymal stem cell markers of hUC-MSCs.**


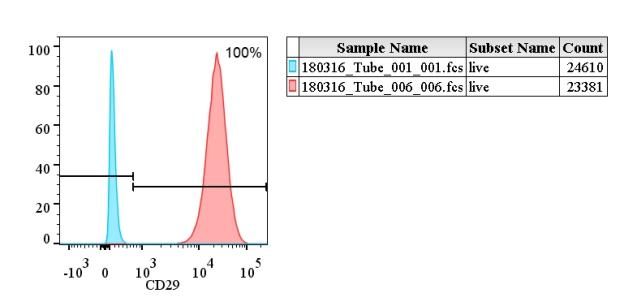

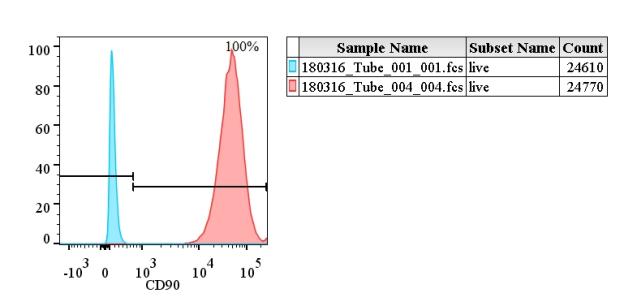
**
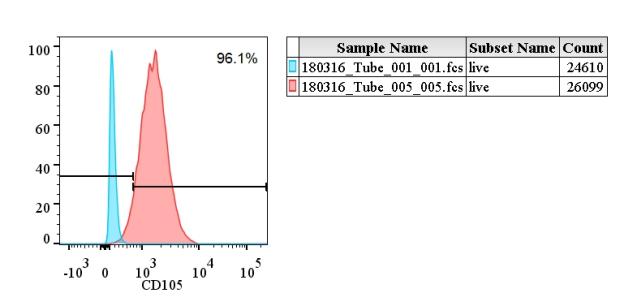

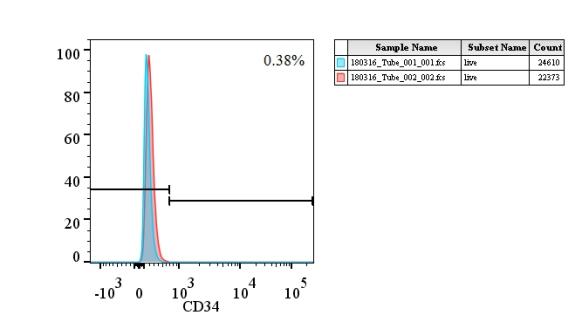
**

**
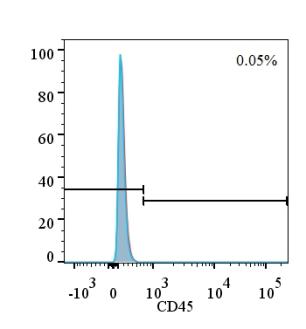
**
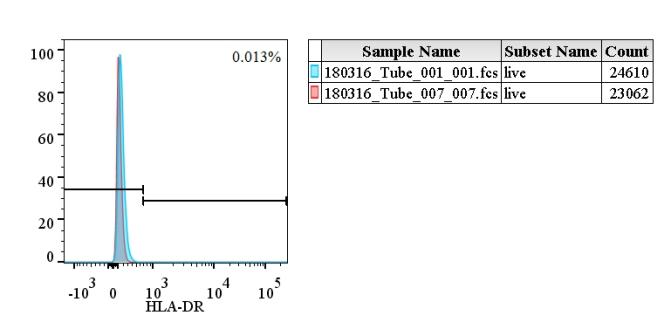


**Figure S2**

**The primitive view of the differentiation of hUC-MSCs.**


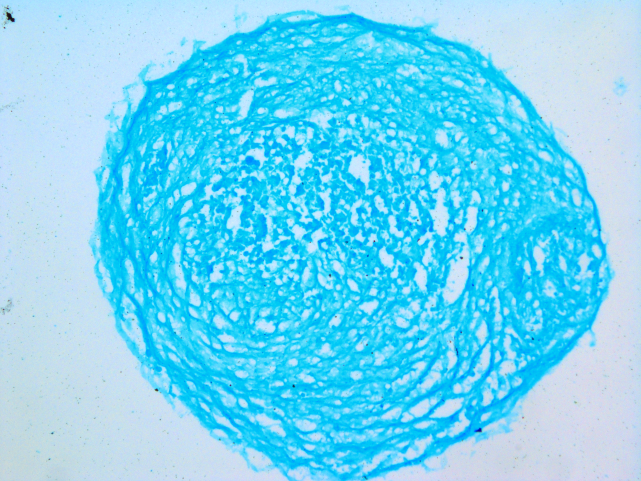

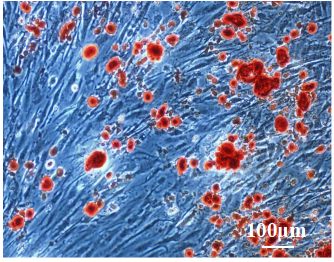

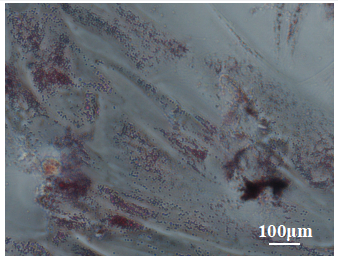


C

B

A

A: hUC-MSCs differentiated into chondrogenic cells. B: hUC-MSCs differentiated into osteogenic cells. C: hUC-MSCs differentiated into adipogenic cells.

**Figure S3**

**The Fluo-3 fluorescence intensity of intracellular Ca^2+^ in HLF-9.**


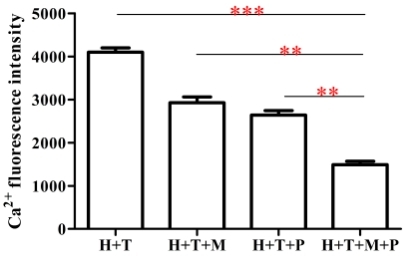


The Fluo-3 fluorescence intensity of Ca^2+^ levels in different group were shown: H+T(A), H+T+M(B), H+T+P(C); H+T+M+P(D). Values are mean ± SEM (n = 3).**p*<0.05; ***p*<0.01;****p*<0.01.

A

**Figure S4**

**The Fluo-3 fluorescence intensity of intracellular Ca^2+^ in NIH3T3.**


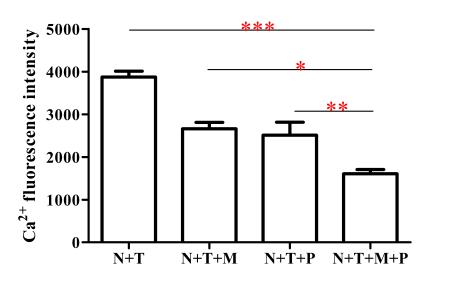


The Fluo-3 fluorescence intensity of Ca^2+^ levels in different group were shown: N+T(A), N+T+M(B), N+T+P(C); N+T+M+P(D). Values are mean ± SEM (n = 3).**p*<0.05; ***p*<0.01;****p*<0.01.

A
